# An Experimental Test of the Information Model for Negotiation of Biparental Care

**DOI:** 10.1371/journal.pone.0019684

**Published:** 2011-05-17

**Authors:** Jessica Meade, Ki-Baek Nam, Jin-Won Lee, Ben J. Hatchwell

**Affiliations:** 1 Department of Animal and Plant Sciences, University of Sheffield, Sheffield, United Kingdom; 2 School of Biological Sciences, Seoul National University, Seoul, Republic of Korea; University of Debrecen, Hungary

## Abstract

**Background:**

Theoretical modelling of biparental care suggests that it can be a stable strategy if parents partially compensate for changes in behaviour by their partners. In empirical studies, however, parents occasionally match rather than compensate for the actions of their partners. The recently proposed “information model” adds to the earlier theory by factoring in information on brood value and/or need into parental decision-making. This leads to a variety of predicted parental responses following a change in partner work-rate depending on the information available to parents.

**Methodology/Principal Findings:**

We experimentally test predictions of the information model using a population of long-tailed tits. We show that parental information on brood need varies systematically through the nestling period and use this variation to predict parental responses to an experimental increase in partner work-rate via playback of extra chick begging calls. When parental information is relatively high, partial compensation is predicted, whereas when parental information is low, a matching response is predicted.

**Conclusions/Significance:**

We find that although some responses are consistent with predictions, parents match a change in their partner's work-rate more often than expected and we discuss possible explanations for our findings.

## Introduction

In species with biparental care the amount of care provided by each parent is often a major source of conflict [1]. The conflict arises because although both parents share the benefit of effort put into raising offspring, provisioning parents pay an individual cost for this effort in terms of decreased survivorship or future fecundity [2], [3]. Thus each parent will seek to increase their own fitness by reducing their reproductive costs at the expense of their partner [4].

Theoretical models seeking the optimal solution to this investment game resolved that for biparental care to be stable, a parent should only partially compensate for a change in partner effort [5]–[9], because full compensation would allow one parent to be exploited by the other. In Houston and Davies' model [6] each parent's optimal investment is fixed over evolutionary time and parents cannot respond to each other in real time. McNamara's negotiation model [7]–[9] proposed that parents negotiate over a behavioural timescale, and is thus more inclusive of potential influences on parental effort. Despite the differences between these two approaches, both models predict similar outcomes, i.e. that there should be incomplete compensation for a change in partner work-rate for biparental care to be a stable strategy.

This key prediction has been empirically tested many times, using a variety of techniques to change the work-rate of one parent, e.g. feather cutting [10]–[14], weighting [15]–[20], manipulation of testosterone levels [21]–[25], playback of chick begging calls [26], [27] or by providing supplementary food [28], whilst monitoring that of the other. The outcome however has been extremely variable [29] resulting in the expected partial compensation [17], [18], [24], full compensation [19]–[23], [28], no response [10]–[14], [15], [16], or a variable response according to sex [14]. Surprisingly, in a few cases parents reacted by changing their effort in the same direction as their manipulated partner (termed ‘matching’) [25]–[27]. Many of these results are inconsistent with the predictions of theoretical models [6]–[9].

There are several potential explanations for these inconsistencies. First, some manipulation techniques may affect perception of partner quality as well as parental effort. If perceived partner quality is reduced by the manipulation, e.g. following handicapping, differential allocation theory [30] predicts that parental effort should decrease because offspring of a poor quality partner are less worthy of investment. Secondly, if one parent is handicapped over a period of several days, chick begging is likely to increase as need increases, and this in turn is likely to affect the provisioning rate of the un-manipulated parent in addition to the change in partner work-rate. Thirdly, the extent to which a parent can increase its provisioning rate in response to the reduced effort of its partner may vary among species or individuals depending on their initial work-rate. Finally, the models assume that both parents have good information about their partner, and about brood value or need, and hence the marginal value of their investment, since begging provides a reliable signal of nestling demand [19], [31], [32]. Recently an alternative model incorporating uncertainty about brood value/need into the negotiation model [7]–[9] has been proposed [33]. This has been referred to as the ‘information model’. Parental information about brood need can vary both between parents and through the nestling phase [33], and if one parent's information about brood need/value is incomplete, they may use their partner's effort as an alternative indicator of how much should be invested in a brood. Parents may therefore integrate information gained from their partner as well as from their nestlings resulting in a range of predicted responses to manipulated partner effort, from partial compensation when they are well informed about brood value/need, through to matching when information on brood value/need is low.

In this study we use a population of long-tailed tits *Aegithalos caudatus* to experimentally test predictions of the information model of biparental care. We address criticisms of previous experiments by adopting Hinde's [26] protocol, using short-term playback of begging calls to manipulate parental work-rate. This method is unlikely to alter perceptions of partner quality and because it is carried out over a short period of time, nestling begging is likely to remain constant. Furthermore, since the compensation and negotiation models predict a reduction in work-rate by the manipulated bird's partner, the potential problem of a ceiling to partner effort is avoided. Importantly the information available to long-tailed tit parents about nestling need is likely to vary systematically through the nestling phase, we focus specifically on need rather than any measure of quality (such as genetic quality) as variation in information on need is easier to assess. During the first 5–6 days after hatching the female broods the chicks for long periods, and if she is present when the male arrives with food he passes it to her and leaves, and the female passes it on to the nestlings. Long-tailed tit nests are domed with only a small entrance hole so the male is likely to gain very little information about brood need at this stage because his direct interactions with chicks are infrequent. After day 6, females rarely brood chicks and both parents lean inside the nest to provision nestlings, thus both parents acquire relatively full, symmetrical information about brood need. As chicks get older they beg with their heads sticking out of the small nest entrance, so at this late stage parents gain information about the need of a subset of chicks, leading to partial, symmetrical information about brood need.

This variation in direct interaction between parents and nestlings across the nestling phase allows us to make predictions based on the information model [33]. Shortly after hatching information is asymmetrical; females having full information and males having partial information, therefore, we predict that males should match an increase in female feeding rate, whereas females should compensate for her partner's increase. During the mid-nestling phase both parents have relatively complete, symmetrical information and we would expect both parents to reduce their work-rate in response to a partner's increase. Late in the nestling phase when both parents have symmetrical, partial information we would expect both parents to increase their own feeding rate to match that of their partners.

In this study we first quantified the amount of information available to parents from direct interactions with chicks at different stages of the nestling phase. We then used playback of begging calls to manipulate the feeding rates of a focal long-tailed tit parent at these different stages of the nestling phase, so that the response of their partner could be examined.

## Methods

### Ethics statement

Blood samples were taken under UK Home Office Licence (project licence holder: BJH, project licence: 4003214, establishment code: 5002509), ringing under BTO licence (BJH SC3770) and the BOU's “Ethics of Ornithological Research (1995)” was adhered to throughout.

### Species and study site

We used data from a long-term study (1994–2010) of a population of long-tailed tits in the Rivelin Valley, Sheffield, UK (53°23′ N 1°34′ W). Routine protocols have been carried out each year. Nests were located by observation of building pairs. Any unringed individuals were caught, uniquely colour-ringed, weighed and bled once nest sites were found. Nests were monitored approximately every second day and lay date, clutch size, hatch date, brood size and fledge date were recorded. Nestlings in accessible nests were weighed, colour-ringed and bled on day 11 (day of hatching  =  day 0). Blood samples were subsequently used to sex individuals. During the nestling phase, nests were watched on alternate days from day 2, typically for a period of 1 hour. For further details of the study site and field protocols see [34]. In addition, we conducted observations and experimental manipulations of provisioning behaviour in 2008–2010 on the same study population, described in detail below.

### Variation in information available through the nestling period

To quantify the relative information available to each parent during the nestling phase we used long-term data, to calculate the mean proportion of each observation period females spent brooding, and the proportion of direct feeds made by both sexes. The analysis is derived from a mean of 6.0±0.32 SE hours of observations per nest at 199 nests.

To quantify the information available to parents during the mid-nestling phase (day 7, i.e. post-brooding), we conducted observations in 2008, where nestlings were removed from their own nest on day 7 and placed into an artificial nest of similar internal diameter to natural nests (artificial nest diameter 7.9 cm *vs* natural nest mean diameter  = 7.6 cm±0.23 SE, *n* = 12), but with a higher roof into which a camera was inserted, so that carers could be filmed as they fed chicks. The artificial nest was necessary because natural long-tailed tit nests are not tall enough to allow the camera to focus on the nestlings. The artificial nest containing chicks was placed in front of the natural nest and left for half an hour for carers to habituate to the new nest. Following this period the nest was filmed for *c*. 60 min from inside using a miniature camera (Sony HQ1), and from outside at a distance of *c*. 1.5 m using a camcorder (Sony Handycam DCR-SR57E) to record the colour rings of carers. We recorded both the number of chicks begging and the number of chicks' heads visible to carers as they fed the chicks, as a proportion of the whole brood, so that we could estimate the information about nestling need available to carers at each feeding visit (*n* = 10 nests).

To quantify the information available to carers during the late-nestling phase (day 10), we filmed nests from outside at a distance of *c*. 1.5 m (*n* = 10 nests). Each nest was filmed for *c*. 60 min using a camcorder (as above) so that the number of chicks begging at the nest entrance could be determined for each visit by a carer. We used a generalized linear mixed effects model, with ‘nest’ as a random factor to compare the information available to carers at mid- and late-nestling phases. The dependent variables were the number of chicks' heads visible (whether the chick was begging or not) as a proportion of the total brood and carer status (mother/father/helper).

### Playback experiments

The aim of the playback experiment was to broadcast begging calls to one parent to increase their provisioning rate, and to record the response of their partner to that increase in work-rate. Based on the results of the pilot study (see Results) playback experiments were performed at three stages of the nestling period: (i) ‘early-stage’ (days 3–5, *n* = 29 nests), when females were typically still brooding chicks; (ii) ‘mid-stage’ (days 7–8, *n* = 23 nests) when females had typically stopped brooding and during which time the parents entered the nest to feed nestlings; and (iii) ‘late-stage’ (days 10–13, *n* = 14 nests) when the chicks were begging at the nest entrance. *N.B.* the sample of broods reduced at successive stages for two reasons: (a) because of nest predation, which occurs naturally at a high rate in this population [35], and (b) because long-tailed tits are cooperative breeders where failed breeders may help at other nests during the nestling period [36] and we excluded all nests where helpers were present (see below).

Working earphones were inserted into experimental nests by threading the adaptor and wire through the back of the nest leaving the earphones inside the nest approximately opposite the entrance hole. This was done one day prior to the early-stage experiment to allow birds to become accustomed to their presence before the experiment started. The earphones remained in the nest until after the mid-stage experiment when they were replaced with a dummy speaker next to the nest entrance. The dummy was replaced with working equipment (50 mm Mylar speaker) 30 min before the late-stage experiment started. The position of speakers at the three experimental stages was such that the playback of begging calls was projected close to the begging chicks (i.e. within the nest at early- and mid-stage, close to the nest entrance at late-stage). Speakers were connected via a custom-made switch box with an amplifier to a walkman (Sony WM-C61). These were concealed in a hide set up at least 5 m from the nest a minimum of 30 min before observations started. Begging calls were recorded from broods of chicks in the field at day 5, 7 and 13 in 2008 using a miniature camera and microphone (Sony HQ1), these begging calls were spliced together using Adobe Audition and recorded onto continuous cassettes. One cassette was made for each nestling phase. Provisioning rates were recorded by direct observation and filmed using a camcorder situated on a tripod at least 1.5 m from the nest. All pairs quickly habituated to the presence of earphones and speakers, and provisioning rates during control observation periods (see below) were typical of those observed at un-manipulated nests [37].

Playback experiments were typically performed to both parents on the same day, with each playback treatment separated by ≥1 hour (mean interval between experiments  = 142.7±6 SE min, *n* = 71 observations at 21 nests; due to access restrictions five pairs of experiments at three nests were carried out on consecutive days). The order in which males and females received playback was alternated within pairs and randomised between pairs. Un-manipulated feeding rates were observed during a control period of *c*. 30 min. Directly after this the focal bird received playback of the appropriate begging calls at its first solo visit to the nest, and the experimental period started immediately after first playback. The focal bird received playback every time it fed the chicks (in the absence of its partner) for the next 30 min. Care was taken to ensure that the target bird's partner could not overhear the playback of begging calls. Playback commenced once the focal bird (in the absence of its partner) reached the nest entrance and ceased once feeding finished. The experimental period continued until the first direct feed by the focal bird after playback ceased. Because of this experimental design both control and experimental periods varied in length (control mean  = 43.1 min, range 28–109 min; experimental mean  = 36.4 min, range 17–61 min; *N.B.* in a few instances the playback period was <30 min because the focal bird's partner was present when they visited the nest towards the end of the experimental period, so no begging calls were broadcast). Provisioning rate was calculated as feeds/hour. Any observations where helpers were present were excluded from our analyses.

### Statistical analysis of playback experiments

We first confirmed that focal birds significantly increased their feeding rates in response to begging calls using paired *t*-tests comparing the provisioning rate of focal birds between the control and experimental periods, for each nestling stage separately. We then used linear mixed effects models to investigate any sex difference in response to playback at the three nestling phases. The response variable was change in provisioning rate (feeding rates during playback minus feeding rate before playback), and the explanatory variables were the nestling phase (early, mid or late), sex of the focal bird, and the focal bird's provisioning rate in the control period. We tested for an interaction between sex and nestling phase. Control feeding rate was included as a covariate because any increase in feeding rate might depend on a parent's initial provisioning rate. Nest was included as a random factor to control for potential non-independence of provisioning rates of parents of the same brood. We then investigated partners' responses to an increase in feeding rate of the focal bird in the same way as described above.

Finally, we investigated whether the relative information available to parents at the mid- and late-nestling phases led to any difference in response to an increase in partner work-rate. We used a linear mixed effects model with change in provisioning rate as the response variable (number of feeds during playback minus number of feeds in control period), with nestling stage (mid/late), partner sex and control feeding rate as explanatory variables. The latter was included to control for differences in feeding rates caused by the difference in nestling age. Nest was included as a random factor to control for non-independence of birds feeding at the same nest.

## Results

### Variation in information available throughout the nestling period

**Figure 1:**
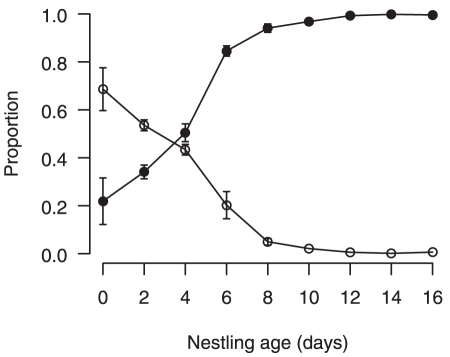
The mean proportion of male feeds direct to nestlings during the nestling period (closed symbols) and the mean proportion of the observation period that the female spends brooding (open symbols). Values are from raw data of control nests (*n* = 202), SE are indicated.

During the early nestling period the information available to male and female parents was asymmetric because females brood until day 5 (Figure 1). As a consequence, females always fed nestlings directly and often took food from males to feed to the chicks, so the proportion of direct male feeds was correspondingly low (Figure 1). After day 6 females spend <20% of their time brooding and ≥85% of male feeds are direct to nestlings. We found a highly significant difference in the number of chicks visible between the mid- and late-nestling periods (χ^2^_1_ = 138.12, *p*<0.001): on days 6–7, an average proportion of 0.81±0.02 SE of the brood was visible to carers, while only 0.19±0.01 of the brood was visible to carers at day 10–12. There was no difference between the number of chicks visible to different types of carer (mother/father/helper, χ^2^_2_ = 0.40, *p* = 0.819). Furthermore, when the number of visible chicks that begged by opening their gapes and extending their necks was compared between the two stages (controlling for ‘nest’) there was still a highly significant difference between the two (mid-stage, mean  = 0.44±0.03 SE, late-stage 0.19±0.01, χ^2^_1_ = 156.25, *p*<0.001).

### Provisioning rates of focal birds

**Figure 2:**
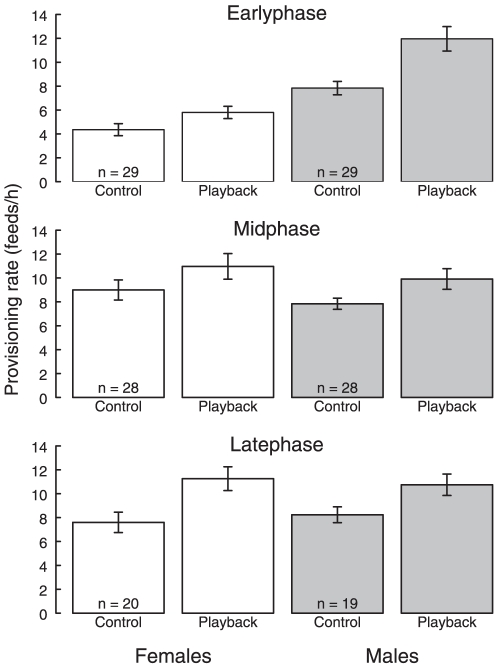
The mean feeding rate of focal females (white bars) and males (grey bars) during the control period and experimental period at all nestling stages. Values are from the raw data. Sample size and SE are indicated.

At all three nestling stages, focal bird provisioning rate increased relative to the control period following playback of begging calls (paired *t*-tests: early-stage, *t*_57_ = 4.6, *p*<0.001; mid-stage, *t*_45_ = 3.8, *P* = 0.001; late-stage, *t*_26_ = 5.0, *p*<0.001; see Figure 2). There was no significant interaction between nestling phase and sex (χ^2^_2_ = 1.35, *p* = 0.265), and there was no significant effect of sex, nestling phase or control feeding rate on the increase in feeding rate of focal birds (see Table 1).

**Table 1:** Results of a linear mixed effects model investigating the change in provisioning rates of focal birds between the control and playback period.

Random effects	Variance		
Nest	<0.001		
Residual	17.909		
Fixed effects	Estimate ± SE	χ^2^	*p*
Intercept	3.397±0.85	-	-
Control rate	0.018±0.11	0.12	0.727
Nestling phase	-	0.14	0.868
Sex	−1.303±0.78	2.69	0.104

### Partner's response to increased provisioning by focal bird

**Figure 3:**
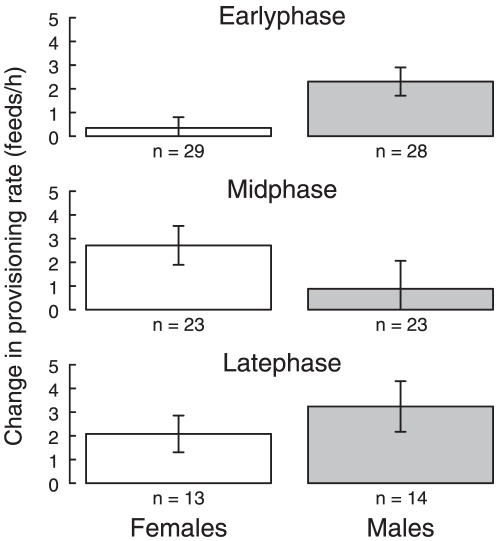
The mean response of partners to an increase in focal bird provisioning rate at each nestling stage. Values are from the raw data. Sample size and SE are indicated.

At all three nestling stages, partners of focal birds significantly increased their provisioning rates (paired *t*-tests: early-stage, *t*_56_ = 3.3, *p* = 0.001; mid-stage, *t*_45_ = 2.4, *p* = 0.017; late-stage, *t*_26_ = 4.0, *p*<0.001; Figure 3). There was a significant interaction between sex and nestling phase (χ^2^_2_ = 4.33, *p* = 0.016, controlling for provisioning rate in control period), and we therefore went on to examine each nestling stage separately. We found that there was a sex difference only at the early-stage (Table 2, Figure 3). Here, males had a greater reaction to an increase in partner work-rate than females, despite the increase in focal female provisioning-rate being smaller than that of focal males (Figure 2). There was no significant change in the provisioning rate of females in response to an increase in male provisioning (paired *t*-test, *t*_28_ = 0.8, *p* = 0.448; Figure 3). At mid- and late-nestling stages there was no sex difference in response, but initial feeding rate during the control period had a significant effect on the magnitude of increase in work- rate, so that parents that fed at lower rates before playback increased their feeding rate to a greater degree (Table 2).

**Table 2:** Results of linear mixed effects models investigating the difference in provisioning rates of partners between the control period and period of playback to focal birds.

	Early-phase			Mid-phase			Late-phase		
Random effects	Variance			Variance			Variance		
Nest	0.060			<0.001			<0.001		
Residual	7.785			19.635			7.992		
Fixed effects	Estimate ± SE	χ^2^	*p*	Estimate ± SE	χ^2^	*p*	Estimate ± SE	χ^2^	*p*
Intercept	1.010±0.78	-	-	6.118±1.61	-	-	6.668±1.71	-	-
Control rate	−0.184±0.14	1.71	0.203	−0.435±0.17	6.93	**0.016**	−0.590±0.19	9.43	**0.011**
Sex	2.403±0.81	8.71	**0.007**	−1.170±1.37	0.72	0.404	1.302±1.16	1.27	0.284

Significant *p*-values are shown in bold.

When the change in feeding rate was compared between mid- and late-nestling stages we found that nestling stage had no effect on the change in provisioning rate (χ^2^_1_ = 0.33, *p* = 0.566). There was no difference between the two sexes (χ^2^_1_ = 0.05, *p* = 0.822) and the increase in provisioning rate was significantly linked to control feeding rate (χ^2^_1_ = 13.86, *p*<0.001).

## Discussion

Taking advantage of the variable information available to long-tailed tit parents through the nestling period we experimentally tested the predictions of the information model [33]. We used playback of begging calls to increase the work-rate of a focal parent. The information model [33] predicts that when parents have full information about brood value/quality they should partially compensate for a change in partner effort. Conversely if a parent has only partial information about brood need they should match changes in partner effort.

We predicted a sex difference in response to focal bird work-rate at the early-nestling stage, when information available to the two sexes is asymmetrical. As predicted, males but not females significantly increased their feeding rate in response to an increase in partner work-rate. This response by males is particularly striking because the increased provisioning-rate of females when exposed to begging playback was relatively small. Thus, a small increase in effort by females elicited a large response by males, whereas a large increase in effort by males in response to playback elicited no significant increase in provisioning rate by females. This result is consistent with the idea that well informed parents will partially compensate for a change in partner effort, whereas less well informed parents will match their partner's effort, however it is important to note that this result could also be caused by constraints on females due to brooding. A study on dark-eyed juncos *Junco hyemalis* suggests that feeding and brooding are competing parental behaviours, although females were still able to significantly increase their feeding rates in the early part of the nestling phase [38].

Once brooding ceases and both parents feed chicks directly, males and females should have symmetrical information, but we predicted a compensatory response at the mid-stage where information is relatively full, and a matching response at the late-stage where information is incomplete. In fact, we found that at both stages both sexes matched an increase in partner work-rate. Furthermore, when we directly compared the change in provisioning rate between mid- and late-stage (controlling for nestling age) we found no difference in response.

The mismatch between the predictions and results at the mid-stage of the nestling period could be a consequence of several factors. First, although our assumptions of the relative information available to parents during the nestling phase make logical sense, very little is known about the actual information that parents gain during provisioning visits, so it is possible that the variation in information that we predict through the nestling phase are not representative of the true information available to the parents. Secondly, assuming our assumptions are correct, although parents had more information available to them at the mid- stage than at the later stage, the information is not ‘complete’ [33]; on average, carers had visual access to 80% of nestlings. Provisioning rules that result in carers matching their partner's effort whenever information is less than 100% would lead to this matching result. Thirdly, long-tailed tits are highly social and usually forage in pairs during the breeding season, or in large flocks during the non-breeding season [39] so coordinated foraging by parents may result in a partner matching the focal parent's increased effort [26], [40], [41], this synchronous provisioning may also be beneficial, potentially reducing nest predation [41] or reducing sibling competition [42]. Similarly, synchronous or strictly alternating parental visits could function as a mechanism for parental negotiation [26], [33]. However, if this was the case one would expect parents to exactly match the feeding rate of their manipulated partner, whereas we find exact matching in only 7% of observations at day 4, and in only 24% of observations at the later nestling stages. It is also theoretically possible that partners could have overheard playback of begging calls to the focal bird. We can discount this possibility because great care was taken to play begging calls to the focal bird only when the other bird was not visible, and the nature of long-tailed tit nesting sites (often isolated bushes, or low level scrub), and highly vocal nature of the birds allowed us to detect the birds' approach long before they arrived at the nest.

The majority of the literature focuses on partner's responses to a *reduction* in focal bird effort and a recent meta-analysis showed that the mean outcome is partial compensation [29]. Very few studies have experimentally *increased* the work-rate of target birds to assess partner response [26]–[28], [43], or indeed increased *and* decreased focal bird work rate in the same system. Our results are consistent with the two studies using playback of extra begging calls [26], [27]; carers matched an experimental increase in the work-rate of other carers. Where focal bird work-rate was experimentally increased by supplemental feeding [28], other carers compensated, but this was probably caused by very high feeding rates of focal birds resulting in rapid satiation of the chicks. This approach also has the important distinction that the increase in focal bird effort is not caused by a perceived increase in chick need. We assumed that an increase in the provisioning rate of the focal parent would act as a passive signal of brood need, to which its partner would respond accordingly. An alternative explanation is that where extra begging calls are used to manipulate a focal parent, because the chicks are perceived to be very hungry, the manipulated parent communicates this extra level of need to other carers at the nest. Communication of chick need by the focal parent combined with an increase in work-rate may have quite a different effect than an increase in work-rate alone. Anecdotally, we noted an increase in vocalisations during playback periods that may have had this function. McDonald *et al*. [27] also reported an increase in the mew call rate of male bell miners targeted with extra begging calls. It is possible, therefore, that parent long-tailed tits may always exhibit a short-term matching response to increases in partner effort elicited by playback of begging calls when not constrained by brooding.

In conclusion, in the current study it is hard to tease apart whether partners of target birds made to work harder by playback of extra begging calls always exhibit short-term matching responses (as in both studies reported thus far; 27,28), or whether the variation in information available to parents at different stages of the nestling phase is insufficient to allow us to detect a difference in response to the manipulation. If elevated provisioning rates were maintained for long periods, one would expect increasing conflict between the signal received from a hard-working partner and that received from a well-fed brood. Therefore the problem may be resolved by conducting similar playback experiments over a longer time period to investigate how information from a partner and information from nestlings is integrated.

## References

[1] Trivers RL, Campbell B (1972). Parental investment and sexual selection.. Sexual selection and the descent of man.

[2] Clutton-Brock TH (1991). The evolution of parental care..

[3] Maynard Smith J (1977). Parental investment: a prospective analysis.. Anim Behav.

[4] Lessells CM, Keller L (1999). Sexual conflict..

[5] Chase I (1980). Cooperative and noncooperative behavior in animals.. Am Nat.

[6] Houston AI, Davies NB, Sibly RH, Smith RM (1985). The evolution of cooperation and life history in the dunnock Prunella modularis.. Behavioural ecology: ecological consequences of adaptive behaviour.

[7] McNamara J, Gasson C, Houston A (1999). Incorporating rules for responding into evolutionary games.. Nature.

[8] McNamara JM, Houston AI, Barta Z, Osorno JL (2003). Should young ever be better off with one parent than with two?. Behav Ecol.

[9] McNamara JM, Székely T, Webb JN, Houston AI (2000). A dynamic game-theoretic model of parental care.. J Theor Biol.

[10] Lifjeld JL, Slagsvold T (1990). Manipulations of male parental investment in polygynous pied flycatchers, *Ficedula hypoleuca*.. Behav Ecol.

[11] Moreno J, Merino S, Potti J, de Leon A, Rodriguez R (1999). Maternal energy expenditure does not change with flight costs or food availability in the pied flycatcher (*Ficedula hypoleuca*): costs and benefits for nestlings.. Behav Ecol Sociobiol.

[12] Slagsvold T, Lifjeld JT (1990). Influence of male and female quality on clutch size in tits (Parus spp).. Ecology.

[13] Whittingham L, Dunn P, Robertson R (1994). Female response to reduced male parental care in birds: an experiment in tree swallows.. Ethology.

[14] Sanz J, Kranenbarg S, Tinbergen J (2000). Differential response by males and females to manipulation of partner contribution in the great tit (Parus major).. J Anim Ecol.

[15] Lozano GA, Lemon RE (1996). Male plumage, paternal care and reproductive success in yellow warblers, *Dendroica petechia*.. Anim Behav.

[16] Schwagmeyer P, Mock DW, Parker GA (2002). Biparental care in house sparrows: negotiation or sealed bid?. Behav Ecol.

[17] Markman S, Yom-Tov Y, Wright J (1995). Male parental care in the orange-tufted sunbird: behavioural adjustments in provisioning and nest guarding effort.. Anim Behav.

[18] Wright J, Cuthill I (1989). Manipulations of sex differences in parental care.. Behav Ecol Sociobiol.

[19] Wright J, Cuthill I (1990a). Biparental care: short-term manipulation of partner contribution and brood size in the starling, *Sturnus vulgaris*.. Behav Ecology.

[20] Wright J, Cuthill I (1990b). Manipulations of sex differences in parental care: the effect of brood size.. Anim Behav.

[21] Stoehr AM, Hill GE (2000). Testosterone and the allocation of reproductive effort in male house finches (Carpodacus mexicanus).. Behav Ecol Sociobiol.

[22] Hunt K E, Hahn TP, Wingfield JC (1999). Endocrine influences on parental care during a short breeding season: testosterone and male parental care in Lapland longspurs (*Calcarius lapponicus*).. Behav Ecol Sociobiol.

[23] Ketterson ED, Nolan V, Wolf L, Ziegenfus C (1992). Testosterone and avian life histories—effects of experimentally elevated testosterone on behavior and correlates of fitness in the dark-eyed junco (*Junco hyemalis*).. Am Nat.

[24] Saino N, Moller AP (1995). Testosterone induced depression of male parental behavior in the barn swallow—female compensation and effects on seasonal fitness.. Behav Ecol Sociobiol.

[25] Hegner RE, Wingfield JC (1987). Effects of experimental manipulation of testosterone levels on parental investment and breeding success in male house sparrows.. Auk.

[26] Hinde CA (2006). Negotiation over offspring care? A positive response to partner-provisioning rate in great tits.. Behav Ecol.

[27] McDonald PG, Kazem AJN, Wright J (2009). Cooperative provisioning dynamics: fathers and unrelated helpers show similar responses to manipulations of begging.. Anim Behav.

[28] Wright J, Dingemanse NJ (1999). Parents and helpers compensate for experimental changes in the provisioning effort of others in the Arabian babbler.. Anim Behav.

[29] Harrison F, Barta Z, Cuthill I, Székely T (2009). How is sexual conflict over parental care resolved? A meta-analysis.. J Evol Biol.

[30] Burley N (1986). The differential-allocation hypothesis: an experimental test.. Am Nat.

[31] Godfray HCJ (1991). Signalling of need by offspring to their parents.. Nature.

[32] Kilner R, Johnstone RA (1997). Begging the question: are offspring solicitation behaviours signals of need?. Trends Ecol Evol.

[33] Johnstone RA, Hinde CA (2006). Negotiation over offspring care-how should parents respond to each others efforts?. Behav Ecol.

[34] Hatchwell BJ, Sharp SP (2006). Kin selection, constraints and the evolution of cooperative breeding in long-tailed tits.. Adv Stud Behav.

[35] Hatchwell BJ, Russell AF, Fowlie MK, Ross DJ (1999). Reproductive success and nest-site selection in a cooperative breeder: effect of experience and a direct benefit of helping.. Auk.

[36] Hatchwell BJ, Russell AF, MacColl ADC, Ross DJ, Fowlie MK (2004). Helpers increase long-term but not short-term productivity in cooperatively breeding long-tailed tits.. Behav Ecol.

[37] MacColl ADC, Hatchwell BJ (2003). Sharing of caring: nestling provisioning behaviour of long-tailed tit, *Aegithalos caudatus*, parents and helpers.. Anim Behav.

[38] Wolf L, Ketterson ED, Nolan V (1990). Behavioural response of female dark-eyed juncos to the experimental removal of their mates: implications for the evolution of male parental care.. Anim Behav.

[39] Hatchwell BJ, Ross DJ, Fowlie MK, McGowan A (2001). Kin discrimination in cooperatively breeding long–tailed tits.. Proc R Soc Lond B.

[40] Lee J-W, Kim H-Y, Hatchwell BJ (2010). Parental provisioning behaviour in a flock-living passerine, the Vinous-throated Parrotbill Paradoxornis *webbianus*.. J Ornithol.

[41] Raihani NJ, Nelson-Flower MJ, Moyes K, Browning LE, Ridley AR (2010). Synchronous provisioning increases brood survival in cooperatively breeding pied babblers.. J Anim Ecol.

[42] Shen SF, Chen HC, Vehrencamp SL, Yuan HW (2010). Group provisioning limits sharing conflict among nestlings in joint-nested Taiwan yuhinas.. Biology Letters.

[43] MacGregor NA, Cockburn A (2002). Sex differences in parental response to begging nestlings in superb fairy-wrens.. Anim Behav.

